# Temporal Trends in Post‐LAAO Surveillance Imaging: Increasing Adoption of CT‐Based Follow‐Up in Contemporary Practice

**DOI:** 10.1111/jce.70418

**Published:** 2026-06-30

**Authors:** Issam Motairek, Ali Dakik, Mohamad Mdaihly, Chadi Tabaja, Bryan Abadie, Mohamed Kanj, Walid Saliba, Oussama Wazni, Wael A. Jaber

**Affiliations:** ^1^ Cleveland Clinic Lerner College of Medicine of Case Western Reserve University School of Medicine, Heart and Vascular Institute Cleveland Clinic Cleveland Ohio USA

**Keywords:** cardiac computed tomography, left atrial appendage occlusion, peri‐device leak, surveillance imaging, transesophageal echocardiography

## Abstract

**Background:**

Surveillance imaging after left atrial appendage occlusion (LAAO) is recommended to evaluate for peri‐device leak and device‐related thrombus, with both transesophageal echocardiography (TEE) and cardiac computed tomography (CT) considered appropriate modalities. Longitudinal trends in surveillance imaging utilization and modality selection have not been well characterized in large, real‐world populations.

**Methods:**

We conducted a retrospective observational study using the TriNetX Research Network, a federated, de‐identified electronic health record database. Adult patients (≥ 18 years) undergoing percutaneous transcatheter LAAO between January 2017 and December 2024 with available imaging data were included. Surveillance imaging was defined as TEE or cardiac CT performed between 45 and 365 days post‐implantation. Annual rates of TEE‐only, CT‐only, and any surveillance imaging were calculated relative to total LAAO volume per year. Temporal trends were assessed using linear regression (*p* < 0.05).

**Results:**

Among 33 267 patients, the proportion undergoing any surveillance imaging increased from 47.2% in 2017 to 66.6% in 2024 (slope +3.1%/year, *p* < 0.001). TEE utilization remained stable (slope +0.11%/year, *p* = 0.68), while CT utilization increased from 2.6% to 22.1% (slope +2.7%/year, *p* < 0.001). The overall increase in surveillance imaging was driven primarily by CT adoption. Approximately one‐third of patients did not undergo any surveillance imaging within 1 year.

**Conclusion:**

Between 2017 and 2024, CT utilization following LAAO increased nearly eightfold while TEE rates remained stable; however, approximately one‐third of patients did not undergo any surveillance imaging within 1 year, revealing a substantial gap in adherence to FDA‐mandated surveillance protocols.

1

Surveillance imaging after left atrial appendage occlusion (LAAO) is recommended 45−90 days after implantation to evaluate for peri‐device leak (PDL) and device‐related thrombus (DRT), with both transesophageal echocardiography (TEE) and cardiac computed tomography (CT) considered appropriate modalities [1]. Despite these recommendations, longitudinal trends in surveillance imaging utilization and modality selection have not been well described in large, real‐world populations. Using a multi‐institutional database, we sought to evaluate temporal trends in post‐LAAO surveillance imaging modality selection.

We conducted a retrospective observational study using the TriNetX Research Network, a federated electronic health record database comprising data from multiple US healthcare organizations, while de‐identifying patients and the sources that provided the data. Our study cohort was restricted exclusively to patients undergoing percutaneous transcatheter LAAO device implantation, identified using device‐specific procedure codes (CPT codes for transcatheter LAAO). Surgical LAAO or ligation procedures (e.g., AtriClip or surgical stapling) were not captured by these codes and are not included in this analysis. Adult patients (≥ 18 years) undergoing LAAO between January 2017 and December 2024 and had longitudinal procedural and imaging data available were included. Given that study subjects are de‐identified, our study was exempt from requiring institutional review board (IRB) approval at the Cleveland Clinic. Surveillance imaging was defined as TEE or cardiac CT performed between 45 and 365 days following device implantation, consistent with guideline‐recommended surveillance windows [1]. Annual rates of TEE‐only, CT‐only, and any surveillance imaging, defined as unique patients receiving at least one surveillance study, were calculated relative to total LAAO procedural volume per year. Temporal trends were assessed using linear regression, with statistical significance set at *p* < 0.05.

A total of 33 267 patients met the study's inclusion criteria. The proportion undergoing any surveillance imaging increased from 47.2% in 2017 to 66.6% in 2024, representing an absolute increase of 19.4% (slope +3.1% per year, *p* < 0.001). TEE utilization remained stable throughout the study period (slope +0.11% per year, *p* = 0.68). In contrast, CT utilization increased significantly from 2.6% in 2017 to 22.1% in 2024, corresponding to an absolute increase of 19.5% (slope +2.7% per year, *p* < 0.001). As a result, the overall increase in surveillance imaging was driven primarily by growth in CT use, while TEE frequency remained unchanged.

These findings reveal that approximately one‐third of patients did not undergo any surveillance imaging within 1 year of LAAO, despite FDA‐approved labeling for WATCHMAN devices mandating imaging at 45 days and 12 months to confirm adequate sealing and exclude DRT before anticoagulation discontinuation [1]. This is particularly concerning given that anticoagulation discontinuation is predicated on imaging confirmation of adequate device sealing and absence of DRT, a complication that occurs in 3%−4% of patients and confers a 3.55‐fold increased stroke risk (95% CI 2.18−5.79) [2]. Importantly, in systematic imaging protocols from pivotal trials, up to two‐thirds of DRT cases were detected more than 6 months after implantation [3]. Similarly, PDL ≤ 5 mm detected at 1 year, but not at 45 days, has been associated with nearly twice the risk of stroke at 5‐year follow‐up (HR 1.94, 95% CI 1.15−3.29) [4]. Collectively, these findings indicate that high‐risk complications may emerge late and remain clinically silent; without surveillance, patients may be transitioned off anticoagulation while carrying elevated thromboembolic risk, highlighting the need for adherence to imaging protocols.

Among patients who did undergo imaging, the nearly eightfold increase in CT utilization from 2017 to 2024 reflects growing recognition of CT's diagnostic advantages, including higher sensitivity for PDL detection compared with TEE (58.8% vs. 34.6%; OR 2.26, 95% CI 1.48−3.44) without compromising DRT detection [5]. This trend may also reflect a shift away from TEE during the COVID‐19 pandemic due to aerosolization concerns. The observed gap in surveillance imaging adherence is unlikely to reflect physician unawareness of post‐procedural protocols, given that percutaneous LAAO is performed predominantly by experienced structural interventionalists and electrophysiologists at high‐volume centers with established follow‐up programs. Rather, failure to complete surveillance imaging more likely reflects patient‐level barriers, including frailty, high comorbidity burden, intercurrent medical illness disrupting follow‐up scheduling, and patient preference against further testing. The LAAO population is, by definition, enriched for patients deemed unsuitable for long‐term anticoagulation, many of whom are elderly and carry substantial competing medical demands. These patient‐level factors—rather than a gap in physician knowledge—likely represent the dominant drivers of nonadherence and warrant targeted investigation in future studies. Additionally, patient‐level factors such as age, sex, baseline stroke risk as estimated by CHA_2_DS_2_‐VASc score, and bleeding risk as estimated by HAS‐BLED score may influence both the decision to pursue surveillance imaging and the modality selected. Future studies with granular registry‐level data should formally evaluate these associations to identify patients at the highest risk of nonadherence.

Study limitations include potential variability in imaging protocols across participating institutions, lack of data regarding imaging indication (routine surveillance vs. symptom driven), and inability to assess clinical outcomes associated with different imaging strategies. Additionally, we could not determine whether individual patients underwent both TEE and CT during the surveillance window. Although our cohort was restricted to percutaneous LAAO procedures using device‐specific coding, we were unable to further stratify by device type (e.g., Watchman vs. Amulet), as the TriNetX platform does not capture device‐brand‐level granularity; this distinction may be clinically relevant given potential differences in post‐implant imaging protocols between devices. Furthermore, the database does not provide reliable institutional‐level metadata to stratify imaging utilization by practice setting (e.g., academic vs. community hospital), which may be an important source of practice variation. Finally, we were unable to formally assess the influence of patient‐level factors such as age, sex, CHA_2_DS_2_‐VASc score, or HAS‐BLED score on imaging utilization in this analysis; future studies should examine these predictors of surveillance adherence.

Between 2017 and 2024, CT utilization following LAAO increased nearly eightfold while TEE rates remained stable; however, approximately one‐third of patients did not undergo any surveillance imaging within 1 year. These findings represent the first characterization of post‐LAAO imaging trends and reveal a substantial gap in adherence to FDA‐mandated surveillance protocols. This gap likely reflects patient‐level barriers rather than physician unfamiliarity with guideline recommendations. Future studies should evaluate the comparative effectiveness of imaging modalities, optimal timing and frequency of surveillance, and outcomes in imaged versus unimaged patients, as well as patient‐level predictors of surveillance nonadherence and the role of practice setting in shaping imaging utilization (Figure 1).

**Figure 1:**
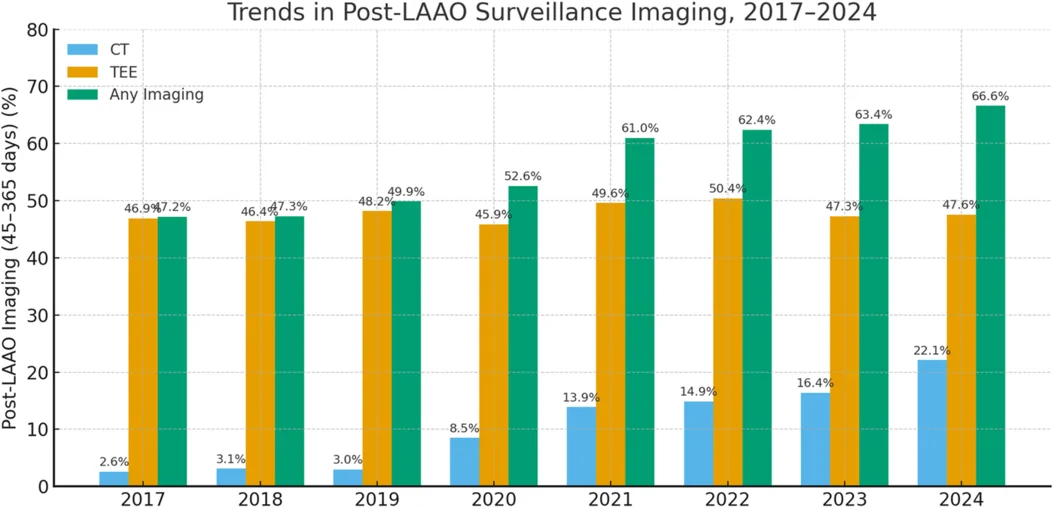
Trends in post‐LAAO surveillance imaging, 2017–2024. Annual proportions of cardiac CT (blue), TEE (orange), and either modality (green) performed 45–365 days after LAAO. Overall imaging increased over time, driven by rising CT use, while TEE remained stable. Data from a multi‐institutional research network.

## Funding

The authors have nothing to report.

## Conflicts of Interest

Wael A. Jaber: Consultant, Boston Scientific and Pfizer. The other authors declare no conflicts of interest.

## Data Availability

The data that support the findings of this study are available on request from the corresponding author. The data are not publicly available due to privacy or ethical restrictions.
